# Recurrent Hypoglycemia Increases Anxiety and Amygdala Norepinephrine Release During Subsequent Hypoglycemia

**DOI:** 10.3389/fendo.2015.00175

**Published:** 2015-11-20

**Authors:** Ewan McNay

**Affiliations:** ^1^Behavioral Neuroscience, University at Albany (SUNY), Albany, NY, USA

**Keywords:** hypoglycemia, insulin, amygdala, anxiety, diabetes, norepinephrine, recurrent hypoglycemia

## Abstract

Recurrent hypoglycemia (RH) is a common and debilitating side effect of therapy in patients with both type 1 and, increasingly, type 2 diabetes. Previous studies in rats have shown marked effects of RH on subsequent hippocampal behavioral, metabolic, and synaptic processes. In addition to impaired memory, patients experiencing RH report alterations in cognitive processes that include mood and anxiety, suggesting that RH may also affect amygdala function. We tested the impact of RH on amygdala function using an elevated plus-maze test of anxiety together with *in vivo* amygdala microdialysis for norepinephrine (NEp), a widely used marker of basolateral amygdala cognitive processes. In contrast to findings in the hippocampus and prefrontal cortex, neither RH nor acute hypoglycemia alone significantly affected plus-maze performance or NEp release. However, animals tested when hypoglycemic who had previously experienced RH had elevated amygdala NEp during plus-maze testing, accompanied by increased anxiety (i.e., less time spent in the open arms of the plus-maze). The results show that RH has widespread effects on subsequent brain function, which vary by neural system.

## Introduction

The opening paragraph of a recent commentary (1) describes the significance of recurrent hypoglycemia (RH) vividly: “[RH] is the limiting factor in the glycemic management of diabetes. It causes recurrent morbidity in most people with type 1 diabetes and many with advanced type 2 diabetes and is sometimes fatal. It impairs defenses against subsequent hypoglycemia and, thus, causes a vicious cycle of recurrent hypoglycemia. The barrier of hypoglycemia generally precludes maintenance of euglycemia over a lifetime of diabetes.”

Hypoglycemia is a common side effect of insulin therapy in both type 1 and type 2 diabetes mellitus (T1 and T2DM). RH, and specifically the impact of RH on the brain (both in actuality and in patients’ worry about such impact), is the biggest obstacle to optimal, intensive insulin therapy aimed at tightly preventing hyperglycemia and restoring normal blood glucose (2–4). The majority of work studying RH and the brain has been in the context of RH-induced hypoglycemia-associated autonomic failure (HAAF): unawareness of and inability to respond to hypoglycemia that can in extremes lead to coma and death, focusing on detection of glucose levels in the ventromedial hypothalamus (VMH) (5–8). However, RH is also clinically associated with marked cognitive and behavioral impairments such as mood swings, impaired judgment and mental flexibility, memory loss, and debilitating anxiety (9–17), many of which are likely to be associated with alterations in amygdala function. Cognitive impairment is especially prevalent during subsequent hypoglycemia, which can have profound consequences: for instance, car accidents are a leading cause of death among diabetic patients, linked to impaired judgment during hypoglycemia (10–13, 18).

Despite this, the neural and cognitive impact of RH has been relatively little studied outside the VMH. Our recent review (19) concluded that there is strong evidence that RH can alter cognitive and neural function, but studies of RH in human beings have often had difficulty in controlling for confounding disease states and/or variable prior history of hypoglycemia (6, 15, 17, 20, 21). In three previous reports that examined RH in rats, we have characterized the impact of RH on subsequent hippocampal function including spatial memory (22, 23) and on mental flexibility, mediated by the prefrontal cortex (24). The rat model of RH used in those studies accurately simulates the effects of RH and HAAF in human beings (22–28), and we have continued to use this model in the present work. Here, we examined the impact of RH on amygdala function. The amygdala plays a key role in anxiety and mood, which are reported to be dysregulated after RH in human beings, and previous studies have shown that similarly to the hippocampus, cognitive processing in the amygdala is limited by glucose metabolism (29, 30), suggesting that RH may alter subsequent amygdala function.

A key finding from the studies of RH and hippocampal function is that the impact of RH varies with acute glycemic state: although hippocampal function is preserved and perhaps enhanced when tested at euglycemia, marked impairment is seen during a subsequent hypoglycemic episode (22, 23), matching symptoms seen in humans. In contrast, mental flexibility and PFC glucose metabolism were impaired after RH even when measured at euglycemia, suggesting that the impact of RH may vary by brain region. That hypothesis is supported by the findings reported here: we show using an elevated plus-maze task that after RH, rats show no change in anxiety or amygdala norepinephrine (NEp) release when measured at euglycemia, but are significantly more anxious during subsequent hypoglycemia, accompanied by elevated amygdala NEp release. NEp release in the basolateral amygdala (BLA) has been widely shown to be a marker for amygdala cognitive modulation (31–33).

## Materials and Methods

### Experimental Timeline

All procedures were approved by the Institutional Animal Care and Use Committee at the University at Albany. 36 male Sprague-Dawley rats (Charles River, Wilmington, MA, USA) were pair housed in enriched conditions (toys, plastic tubing, paper cups, etc.) From 11 weeks of age, rats are handled daily for a minimum of 10 min; this reduces stress hormone release at the time of testing to baseline levels (22). At 13–14 weeks, animals underwent stereotactic implantation of a microdialysis guide cannula (CMA12, CMA/Microdialysis) into the left BLA, then 1 week of single-housed recovery with close monitoring and continued handling. At 14–15 weeks, animals were treated with either i.p. insulin or i.p. saline once daily for 3 days, then tested on the fourth day, humanely killed, and samples taken for analysis. At the start of treatment, animals were randomly assigned to either control or RH conditions; on the day of testing, animals were randomly assigned to either hypoglycemic or euglycemic conditions. This created four groups with between 8 and 10 animals in each group in a 2 × 2 factorial design.

### Surgical Procedures

Rats were anesthetized with 5% isoflurane. Standard sterile stereotaxic surgical procedures were used as described previously (34–36) to implant the microdialysis guide cannula, secured in place with acrylic cement and two screws, and a dummy stylet was inserted. Rats recovered in a heated chamber and returned to their home cages once they had regained consciousness and full motor control. Animal recovery was monitored for 3 days. Rimadyl once daily was used for post-surgical analgesia. Correct cannula placement was confirmed visually in all animals at the time of tissue extraction by locating the tract path created by the cannula which terminated in the BLA: all animals had correctly placed cannulae.

### Microdialysis

As published (22, 30, 37–40): a fresh probe was inserted, and animals were acclimated for 2 h prior to testing. The dialysis membrane was 1 mm. Rats moved freely, avoiding any confound from restraint stress. Probes were perfused with an artificial extracellular fluid [aECF; composition in millimolar: 153.5 Na, 4.3 K, 0.41 Mg, 0.71 Ca, 139.4 Cl, 1.25 glucose, buffered at pH 7.4 (40)] at 1.5 μL/min. Microdialysis samples were frozen for later NEp analysis.

### Hypoglycemia

Hypoglycemia was induced with 10 U/kg insulin (Humulin, Eli Lilly) given i.p. to animals made hypoglycemic for the first time, or either 8 or 6 U/kg (because of reduced counter-regulation) to RH animals (22, 23, 41); control animals receive volume-matched sterile saline.

### RH Model

The model used here (3 h of moderate hypoglycemia on each of three consecutive days, followed by testing on the fourth day) has been validated as accurately recreating adaptation seen in human patients with RH. Animals received i.p. insulin (Humulin, Eli Lilly) at 10, 8, and 6 U/kg over the 3 days, with reduction in doses compensating for reduced counterregulation due to HAAF. This reliably produced 2–3 h between 40 and 50 mg/dL plasma glucose; any animal not spontaneously recovering after 3 h was returned to normoglycemia using i.p. glucose. Results from this model closely track those obtained in a 16-month study using once-weekly 3-h hypoglycemia (23), matching the experience of human patients receiving insulin therapy (42–45). In our previous studies, data from T1DM and non-diabetic animals did not differ (22), supporting RH studies in non-diabetic animals to avoid confound from disease-state variables; these data were consistent not only for behavior but also for hippocampal metabolism (22) and synaptic electrophysiology (23). During hypoglycemia, animals were randomly sampled via thigh prick to confirm target hypoglycemia.

### Performance Variable Controls

In studies to date, RH in our model has not impaired or reduced motor activity, visual acuity, or, e.g., motivation: for example, RH animals make the same number of maze-arm choices (22, 23), have the same latency to seek reward (24), and perceive both visual and textural stimuli as well as or better than control animals (24). We confirmed that RH had no measureable effects on motor performance, motivation, or sensory acuity using small separate cohorts of animals, treated identically to those reported here and tested on a simple Y-maze alternation task.

### Elevated Plus-Maze Testing

With microdialysis throughout, animals were placed into the center of a four-arm plus-maze constructed from opaque black plexiglas and allowed to explore freely for 10 min, then returned to home cages. Two, opposing, arms of the four-arm maze had no walls; the other two arms had 20 cm-high walls. Animals spend the majority of time in a closed arm with periodic forays to explore the open arms and/or cross the maze center. Increased time spent in the open arms is taken as a measure of decreased anxiety. Microdialysis is performed in the BLA, where this task is mediated (46–49).

### Sample Analysis

Microdialysis samples were measured for NEp using HPLC on an ESA Coulochem III.

### Data Analysis

Data were analyzed in GraphPad Prism using a two-way ANOVA design with RH (or control) and acute glycemic state as the two factors. Where significant main effects were seen, *post hoc* group comparisons using Tukey’s multiple comparisons test identified specific inter-group differences.

## Results

### Plus-Maze Performance

**Figure 1:**
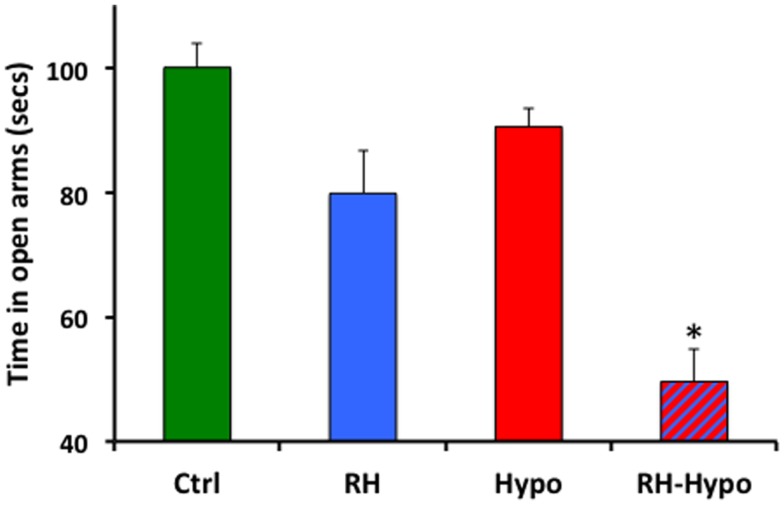
**Animals in the RH-hypo group spent significantly less time in the open arms of the plus-maze, on average, than did animals in other groups**. * indicates significant difference vs all other groups, *p* < 0.001. This is interpreted as increased anxiety in the RH-hypo animals. *N* = 8 for control and hypo groups, and *N*  = 10 for RH and RH-hypo groups.

Both RH treatment and acute glycemic state had significant effects on anxiety, as measured by time spent in the open arms during plus-maze testing (both *p* < 0.001). As shown in Figure 1, *post hoc* comparisons showed that animals in the RH-hypo group spent significantly less time in the open arms than animals in all other groups, indicating increased anxiety (all *p* < 0.001). No other inter-group comparisons showed significant differences. Importantly, no effect of RH was seen on number of center-crossings, supporting the conclusion from our performance control experiments that this difference in open-arm time was not the result of altered motor function or motivation in the RH-hypo group.

### Amygdala Norepinephrine Release

**Figure 2:**
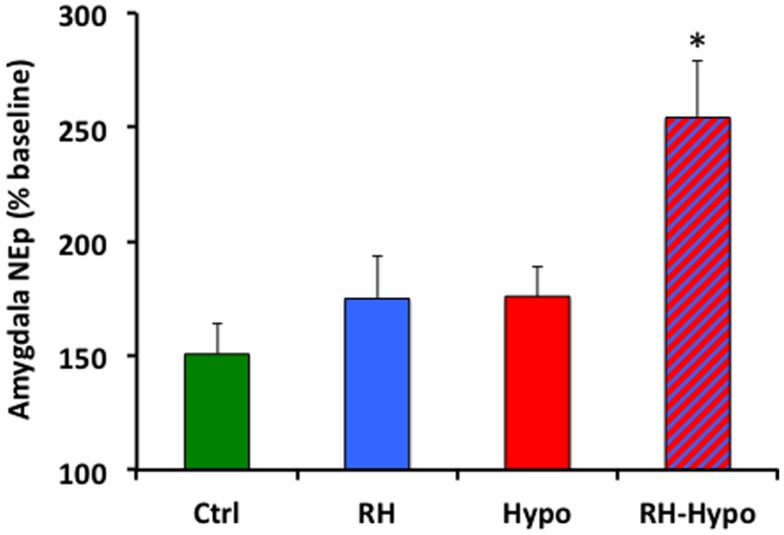
**Animals in the RH-hypo group had significantly higher levels of NEp in microdialysis samples from the basolateral amygdala during elevated plus-maze testing, on average, than did animals in other groups**. * indicates significant difference vs all other groups, *p* < 0.05. This is interpreted as increased anxiogenic processing in the amygdala of animals in the RH-hypo group. *N*  = 8 for control and hypo groups, and *N*  = 10 for RH and RH-hypo groups.

Mean microdialysis sample NEp concentration during the plus-maze testing is shown in Figure 2, reported as a percentage of baseline NEp concentration (with baseline defined as the mean of the three samples immediately prior to placement on the plus-maze; absolute baseline NEp levels did not vary across groups). Consistent with the behavioral data, significant effects of both treatment and glycemic state were seen (both *p* < 0.05) in which *post hoc* comparisons revealed to be due to a significantly increased NEp concentration in samples from RH-hypo animals compared to those in all other groups (all *p* < 0.05, no other significant inter-group differences).

## Discussion

Our data are both consistent with, and extend, previous studies that have examined the impact of RH on subsequent cognitive and neural function: when tested during a hypoglycemic episode, animals with prior RH treatment showed both heightened anxiety and increased amygdala activity, assessed by NEp levels in the BLA.

Our previous work (22–24, 50) suggested that neural adaptations seen following RH might be maladaptive during subsequent hypoglycemia. This is consistent with the clinical experience of patients, where RH is associated with, e.g., increased risk of death while driving. Patients also report symptoms, including alterations in mood and anxiety, that are suggestive of altered emotional processing subsequent to RH: the present data support these reports and suggest that amygdala responsiveness to an aversive stimulus such as exposure on an elevated, open platform may be increased when hypoglycemic after RH. Because amygdala cognitive processing causes increased local glucose metabolism (29), meeting the metabolic requirements of such increased amygdala activation might further diminish the brain’s ability to function optimally at times of reduced glucose availability.

On the other hand, it is possible to speculate (based on the small amount of data presented here) that increased anxiety, fear, or similar emotional arousal might be at least somewhat adaptive in that it could serve as a signal for danger at times of hypoglycemia, alerting the patient to an acute need for fuel. One common effect of RH is diminished release of stress hormones during hypoglycemia [known as HAAF; (5, 51, 52)]: increased amygdala responsiveness caused by RH could, perhaps, be a beneficial adaptation that would oppose and attenuate reduced awareness of hypoglycemia. Stress hormones including epinephrine and glucocorticoids are key modulators of cognitive function, and especially of improved performance at times of moderate stress (31, 53–55), effects that are transduced via the amygdala; it is hence possible that an increase in amygdala responsiveness may be adaptive in acting to positively modulate other brain regions [in particular, the hippocampus; (55–57)] even when systemic hormone release is attenuated. Importantly, though, one study that examined amygdala metabolism in humans, during hypoglycemia, found that in contrast to the present findings fluorodeoxyglucose uptake was better maintained in the amygdala of aware vs unaware patients (58); this is in contrast to our data that suggest increased amygdala activity in the RH animals which would be expected to correspond to hypoglycemia-unaware patients. Although there are significant methodological differences as well as a species difference between the studies, this finding does constrain the ability to generalize from the small dataset presented here. It is also true that stress-related hormones, particularly epinephrine, are released when hypoglycemic but such release diminishes after RH: thus, the enhanced anxiety in the RH-hypo group observed here is somewhat paradoxical and the amygdala’s response to stress hormones under such conditions may repay further study.

The role of hypoglycemia-associated hormone release in alteration of cognitive processes subsequent to RH merits further attention. One of the best supported molecular causes of HAAF, in the VMH, is hypoglycemia-associated GC release, and several studies show that prevention of GC signaling in the VMH during RH prevents HAAF (7, 59–61). Similar causality may be involved in the cognitive impact of RH: glucocorticoid receptors (GRs) are expressed at high levels in the hippocampus (62, 63), and GCs have been extensively shown to mediate hippocampal damage from metabolic stressors (such as hypoglycemia): specifically, GCs exacerbate damage from inadequate glucose supply (64–66) and are linked to excitotoxic cell death following severe hypoglycemia. Conversely, when fuel supply is adequate, GCs enhance hippocampal memory and glutamate release (54, 55, 67): this pattern closely matches the impact of RH on hippocampal function seen in our previous work (22, 23). We did not measure GC levels either systemically or centrally during these studies, but future work should consider including such measurements.

Taken together with our previous studies, the findings here indicate that RH affects multiple neural systems and brain structures, with the impact of RH varying by region and system. For instance, during subsequent euglycemia, RH enhances hippocampal memory (22, 23), impairs mental flexibility processes in the prefrontal cortex (24), but does not affect performance in an elevated plus-maze test of anxiety (present data). The ability of a rodent model of RH to accurately mimic many of the cognitive effects seen in human patients after RH suggests that this is an appropriate system for further studies aimed at identifying the molecular mechanisms transducing the cognitive, neural, and metabolic impact of RH, with a goal of identifying appropriate therapeutic approaches to prevention and intervention.

## Conflict of Interest Statement

The author declares that the research was conducted in the absence of any commercial or financial relationships that could be construed as a potential conflict of interest.
